# Sinopharm’s BBIBP-CorV Vaccine and ChAdOx1 nCoV-19 Vaccine Are Associated with a Comparable Immune Response against SARS-CoV-2

**DOI:** 10.3390/vaccines10091462

**Published:** 2022-09-03

**Authors:** Ahmed Samir Abdelhafiz, Asmaa Ali, Mahmoud M. Kamel, Eman Hasan Ahmed, Douaa M. Sayed, Rania M. Bakry

**Affiliations:** 1 Department of Clinical Pathology, National Cancer Institute, Cairo University, Kasr Al-Aini Street, El-Khalig Square, Cairo 11796, Egypt; 2 Department of Pulmonary Medicine, Abbassia Chest Hospital, MOH, Cairo 11517, Egypt; 3 Department of Laboratory Medicine, School of Medicine, Jiangsu University, Zhenjiang 212013, China; 4 Department of Clinical Pathology, South Egypt Cancer Institute, Assiut University, Assiut 71515, Egypt

**Keywords:** COVID-19, SARS-CoV-2, Egypt, ChAdOx1 nCoV-1, BBIBP-CorV

## Abstract

Coronavirus disease 2019 (COVID-19) has affected millions of people worldwide. During the early stages of vaccination in Egypt, the ChAdOx1 nCoV-19 and BBIBP-CorV vaccines were the most distributed. The aim of this study was to compare the immune responses and short-term efficacies of these two vaccines. We recruited adults who received two doses of either vaccine. Samples were collected after the first dose of ChAdOx1 nCoV-1 and after the second dose of both vaccines. Antibodies against SARS-CoV-2 antigens were measured using LABScreen™ COVID Plus kits, and cell-mediated immune responses were assessed using flow cytometry. Of the 109 recruited subjects, 60 (55%) received the ChAdOx1 nCoV-19 vaccine, and the remainder received the BBIBP-CorV vaccine. The total antibody level did not significantly differ between the two groups. The level of the anti-spike subunit 2 (S2) antibody was significantly higher in the ChAdOx1 nCoV-19 group. The percentages of both total T cells and B cells were unaffected by the type of vaccination. However, the ChAdOx1 nCoV-1 vaccine was significantly associated with a higher percentage of CD8+ cells. The vaccines did not significantly differ in the number or severity of infections postvaccination. None of the participants were admitted to the hospital or died of COVID-19 infection. In conclusion, the BBIBP-CorV vaccine is associated with an immune response and protection against infection that is comparable to that of the ChAdOx1 nCoV-1 vaccine. Follow-up is needed to study the long-term protective effects of both vaccines. Inactivated vaccines are easier to manufacture in developing countries and their limited side effects may lead to better economic benefits by limiting the number of absences from work.

## 1. Introduction

Coronavirus disease 2019 (COVID-19), caused by the severe acute respiratory syndrome coronavirus 2 (SARS-CoV-2), has affected millions of people worldwide. Although the disease is mild in most cases, it progresses to a severe form in some patients and may cause mortality, especially among the elderly and people with comorbidities [1,2]. Few drugs have proven to be effective against the virus, especially during the early phase of the pandemic. Hence, research on vaccines has become a priority, especially for medical personnel and high-risk groups [3].

The rapid development of vaccines has become a global collaborative effort, and about 300 have been developed/tested worldwide [4]. These efforts led to the World Health Organization’s emergency approval of nine COVID-19 vaccines until December 2021, with others still being examined [5]. Interestingly, this pandemic witnessed the approval of mRNA-based vaccines for the first time [6]. Besides this novel type, several other vaccines, including formulations based on replicating viral vectors or virus-like particles, nonreplicating viral vectors, protein subunits, and inactivated vaccines, have also been investigated [7].

Although several vaccines have been approved in different countries, the short- and long-term immune responses associated with different vaccine types are still debatable. Oxford/AstraZeneca’s adenovirus-vectored vaccine ChAdOx1 nCoV-19 and Sinopharm’s inactivated virus vaccine BBIBP-CorV, which is comprised of virus particles grown in culture, were commonly distributed during the early stages of vaccination in Egypt. Due to their wide usage, their comparative efficacies and side effects were highly debated by the scientific community, healthcare personnel, and the public. Many groups assumed that the inactivated vaccine provides less protection than the adenovirus-vectored vaccine. We aimed to evaluate this hypothesis and compare the humoral and cell-mediated immune responses elicited by these vaccines as well as their short-term efficacies in preventing COVID-19 infection and its associated complications.

## 2. Subjects and Methods

### 2.1. Subjects

This study included adults who received two doses of either Oxford–ChAdOx1 nCoV-1 or the BBIBP-CorV vaccine. Adults who received other types of vaccines, have cancer, or currently receive immunosuppressive drugs were excluded. The sample size was determined using the using Minitab 17.1.0.0 for windows (Minitab Inc., 2013, State College, PA, USA). The target population number that accepted to take one of the vaccines in Egypt exceeded 20,000. Based on the previously published data [8,9], the average immunogenicity was expected to exceed 90%. To achieve a 5% margin of error of the study at 90% confidence limits, the minimum calculated sample size was 97 participants. Sampling was carried out until a representative sample of both vaccines was reached. Six ml of peripheral venous blood was collected from participants on EDTA and serum tubes under complete aseptic conditions 14 days after the first dose of the Oxford–ChAdOx1 nCoV-1 vaccine. Another sample was collected 28 days after the second dose of both vaccines. Samples were collected with the same procedure and timing in both groups. Participants filled out an information sheet about their demographic, COVID-19-related, and vaccine-related data, including the type of vaccine and its side effects. Three months after the second dose, the participants were contacted for information about COVID-19 infection and its potential complications after this dose. The Ethical Committee of the South Egypt Cancer Institute, Assiut University, approved this study, and all subjects provided informed consent before participating in the study.

### 2.2. Methods

#### 2.2.1. Antibody Detection

The serum was separated from the blood samples to measure SARS-CoV-2 antibodies using LABScreen™ COVID Plus kits (Catalog Number LSCOV01). Dilution was performed for both control and patients’ samples as follows: (a) First: both the control serum (Catalog # LS-NC and LSCOV-PC) and the test sample were prepared by adding 2μL of serum to 17 μL of 1XPBS; (b) Followed by adding 1 μL of 0.02M EDTA solution to diluted serum from the previous step, which was used for analysis. The SARS-CoV-2 antibodies present in the serum bound to the purified SARS-CoV-2 antigens immobilized on microbeads in the kit. Subsequently, bead–antibody complexes were fluorescently tagged with R-phycoerythrin-conjugated goat antihuman immunoglobulin G (IgG). The results were analyzed on the Luminex^®^ 100/200™ instrument (Austin, TX, USA, serial number LXSD 13107003) using the Luminex xPonent^®^ 4.2 software (Austin, TX, USA).

For a given serum, the value for Positive Control (PC)/Negative Control (NC) beads should be greater than 50. A lower value may be due to an extremely high NC bead background value for the test serum or a low signal from the secondary antibody or the LABScan3D flow analyzer. We accepted the data when the PC/NC was more than 50. Regarding internal validation, both the calibration and validation of Luminex^®^ 100/200™ have been performed by calibration and verification kits: calibration kit cat No (LX200-CAL-K25) and verification kit Cat No (LX200-CON-K25). The agreement of the method has been performed by collecting the serum and plasma of 30 positive cases and 30 negative cases for COVID-19 infection tested by RT-PCR after more than 15 days from the date of the result. The presence of neutralizing antibodies using the CDC antibody neutralization test was performed. The COVID-19 status of each positive patient was confirmed. Negative COVID-19 cases were also confirmed. All tested samples (negative and positive) were performed by the two methods (CDC neutralization kit and Labscreen COVID plus kit, one lambda). The Positive Percent Agreement (PPA) was 100% for the serum tested on the LS100; the Negative Percent Agreement (NPA) was 100% for the serum tested on the LS3D. The PPA was 100% for the plasma tested on the LS100; the NPA was 100% for the plasma tested on the LS3D.

#### 2.2.2. Flow Cytometry

##### Monoclonal Antibodies

The expression of T cell and B cell lineage receptors was assessed by phenotyping using the following monoclonal antibodies (all purchased from BD, CA): antihuman FITC-conjugated antiCD3, PE-conjugated antiCD4, PerCP-conjugated antiCD8, APC-conjugated antiCD19, PC7-conjugated antiCD38, and the corresponding isotype controls. According to the manufacturer’s instructions, peripheral blood mononuclear cells were stained with these antibodies at concentrations titrated for optimal staining. The multicolor staining of monoclonal antibodies was carried out in the following panel: CD3/CD4/CD8/CD19/CD38.

##### Flow Cytometric Analysis

Flow cytometry was performed on the Canto II Flow Cytometer (BD Biotec, CA). Fifty-thousand events were acquired. The gate around the lymphocyte cell regions was drawn to include all lymphocyte cells (R1) based on forward scatter/side scatter and excluded debris, dead cells, platelet aggregates, and myeloid cells. The cells were evaluated by applying the fluorescence minus-one color compensation strategy. The gating strategy used for identifying different lymphocyte populations was as follows:

CD3 + cells were identified. Subsequent phenotypic analysis identified
CD4 + and CD8 + T cell subsets based on CD4 and CD8 expression, respectively. Gated cells were also reanalyzed for expressions of CD19 and CD38 to identify B cells.

All expressions were estimated by the percentage of CD4 and CD8 cell subsets from T lymphocytes. The cutoff of positivity was resolved by isotypic control.

### 2.3. Statistical Analysis

The data were collected using an Excel sheet and statistically analyzed using Minitab 17.1.0.0 for Windows (Minitab Inc., 2013, State College, PA, USA). The continuous data are presented as mean, standard deviation, median, and interquartile range (IQR), while the categorical data are presented as number and percentage. The normality of the data was examined using the Shapiro–Wilk test. The independent *t*-test was used to compare two groups of continuous data while the chi-squared test was used to compare two or more groups of categorical data. The paired *t*-test was used to compare two means before and after an intervention. General linear models with stepwise elimination methods were applied to detect factors that affected cell-mediated immunity, while logistic regression models were used to detect factors implicated in the development of effective antibody responses. All tests were two-sided, and *p* < 0.05 was considered statistically significant.

## 3. Results

### 3.1. General Characteristics of the Participants

One hundred and nine people participated in the study: 60 (55%) received the ChAdOx1 nCoV-19 vaccine; the remaining received the BBIBP-CorV vaccine. Both groups were sex-matched (*p* = 0.83). However, the participants who received the BBIBP-CorV vaccine were significantly older and had comorbidities (*p* < 0.001 for both), in addition to having a history of significant positive contact with COVID-19 patients (*p* < 0.03) (Table 1).

### 3.2. Humoral Immune Response to SARS-CoV-2 Vaccines

**Figure 1:**
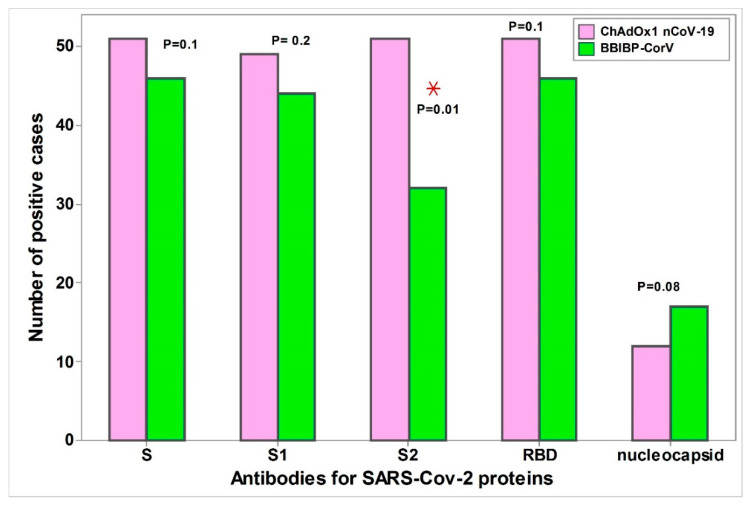
Humoral immune response after the second doses of the ChAdOx1 nCoV-1 and the BBIBPCorV vaccine. * Test of significance: chi-squared test, *p* < 0.05 was considered significant. Cutoff of MFI for Anti-Spike was 7500, Anti-Spike S1 was 4000, Anti-Spike RBD was 3500, Anti-Spike S2 was 1900, Anti-nucleocapsid was 3500. * Represents the Statistically significant values.

After the second dose of both vaccines, the frequency of positive antibodies against different SARS-CoV-2 proteins matched between the two vaccine groups with insignificant differences, except against S2 proteins, where the number of positive subjects having this antibody was significantly higher in the ChAdOx1 nCoV-19 vaccine group (*p* = 0.01, Figure 1). The frequency of positive antibodies against different SARS-CoV-2 proteins after the first dose of ChAdOx1 nCoV-19 vaccine was significantly lower than that after its second dose or even after the second dose of the BBIBP-CorV vaccine (*p* < 0.05, Table 2).

**Figure 2:**
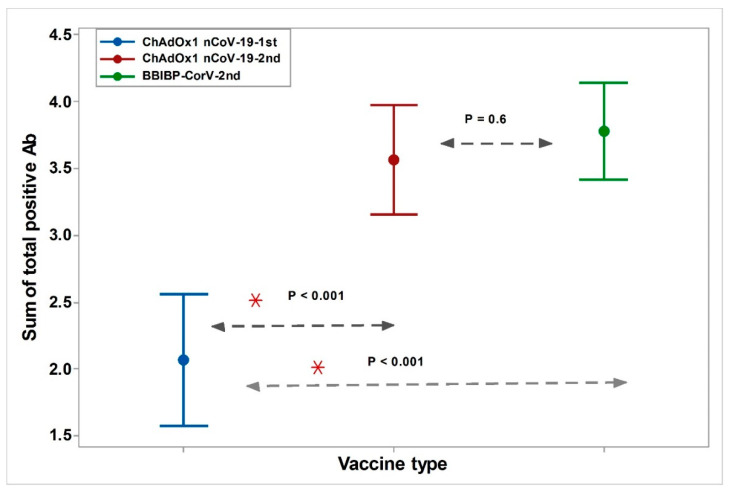
Cumulative total positive antibodies against SARS-CoV-2 proteins after the first and second doses of ChAdOx1 nCoV-19 and the second dose of BBIBP-CorV. * Test of significance: one-way analysis of variance with the Tukey method, *p* < 0.05 was considered significant. ChAdOx1 nCoV-19 1st: The first dose of ChAdOx1 nCoV-19 vaccine. ChAdOx1 nCoV-19 2nd: The second dose of ChAdOx1 nCoV-19 vaccine. BBIBP-CorV2nd: The second dose of BBIBP-CorV vaccine. Cutoff of MFI for Anti-Spike was 7500, Anti-Spike S1 was 4000, Anti-Spike RBD was 3500, Anti-Spike S2 was 1900, Anti-nucleocapsid was 3500. * Represents the Statistically significant values.

Moreover, the cumulative number of positive antibodies after the first dose of the ChAdOx1 nCoV-19 vaccine (number of positive antibodies per participant) was significantly lower than that after the second dose of both vaccines (*p* < 0.001, Figure 2). However, the cumulative number of positive antibodies after the second dose did not differ significantly between the two vaccine groups (*p* = 0.6).

**Figure 3:**
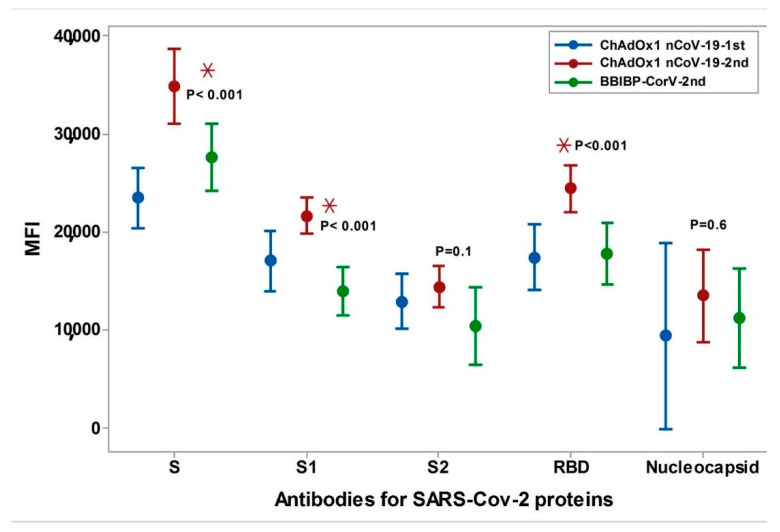
Comparison of the different antibody levels after the first dose of ChAdOx1 nCoV-1 vaccine, second dose of ChAdOx1 nCoV-1, and after the second dose of BBIBP-CorV vaccine. ChAdOx1 nCoV-19 1st: the first dose of ChAdOx1 nCoV-19 vaccine, ChAdOx1 nCoV-19 2nd: The second dose of ChAdOx1 nCoV-19 vaccine, BBIBP-CorV2nd: The second dose of BBIBP-CorV vaccine, MFI: Mean Fluorescent Intensity. Cutoff of MFI for Anti-Spike was 7500, Anti-Spike S1 was 4000, Anti-Spike RBD was 3500, Anti-Spike S2 was 1900, Anti-nucleocapsid was 3500. * Represents the Statistically significant values.

Figure 3 shows a comparison of the different antibody levels after the first dose of the ChAdOx1 nCoV-1 vaccine, the second dose of ChAdOx1 nCoV-1, and the second dose of the BBIBP-CorV vaccine.

### 3.3. Cell-Mediated Immune Response after Vaccination and Factors Affecting its Activation

The univariate analysis revealed that the vaccination type insignificantly affected the percentages of T cells and B cells (Table 3). However, the ChAdOx1 nCoV-1 vaccine was associated with a significantly higher percentage of CD8^+^ cells than the BBIBP-CorV vaccine (*p* = 0.02).

As shown in Table 4, a multivariate general linear model was applied to detect factors that affected both T cell and B cell percentages.

### 3.4. Side Effects of Vaccination

About half of the participants who received the BBIBP-CorV vaccine did not experience any side effects. Moreover, the side effects were significantly milder compared with the moderately severe side effects after the ChAdOx1 nCoV-1 vaccine (*p* < 0.001). The range of the total number of reported side effects per subject was higher in the ChAdOx1 nCoV-1 group (0–4) than in the BBIBP-CorV group (0–3) (*p* = 0.01). Fever and body pain were frequently reported in the ChAdOx1 nCoV-1 group (*p* < 0.001 and 0.01, respectively), while pain at the injection site was the common side effect after the BBIBP-CorV vaccine (*p* = 0.02, Table 5).

### 3.5. Short-Term Protective Effect of Vaccination and Factors Affecting Postvaccine Infection

The post-vaccine protection did not differ significantly between the ChAdOx1 nCoV-1 (58.4%) and the BBIBP-CorV groups (55.1%) (*p* = 0.26). Additionally, post-vaccine infection severity did not differ significantly between the two groups (*p* = 0.3). The median (IQR) of the duration of protection was 1 (1–2) month for the ChAdOx1 nCoV-1 group and 2 (1–3.5) months for the BBIBP-CorV group (*p* = 0.01, Table 6).

## 4. Discussion

Understanding the immune response after vaccination against SARS-CoV-2 is necessary to predict protection against reinfection and help plan better vaccination programs. Our data show that the ChAdOx1 nCoV-1 and BBIBP-CorV vaccines did not differ significantly in the seroprevalence of elicited antibodies against different SARS-CoV-2 antigens.

The SARS-CoV-2 genome encodes 4 structural and 16 nonstructural proteins. Among the structural proteins, the spike (S) and nucleocapsid proteins are the main immunogens, and both are produced in high quantities during COVID-19 infection [10]. The S protein is used by the virus to enter cells and is composed of two subunits: S1, containing the RBD, which binds angiotensin-converting enzyme 2 receptors, and S2, which mediates the fusion between the viral envelope and the cell membrane [11]. A significant strength of our method is that it detected antibodies against different SARS-CoV-2 antigens. Interestingly, there was no significant difference between the two vaccination groups in the number of positive antibodies, anti-S1, or anti-RBD antibodies.

Our data are quite different from those reported in Jordan, where the seroconversion rate for anti-RBD antibodies was significantly higher in the ChAdOx1 nCoV-1 than in the BBIBP-CorV vaccine group [10]. This difference could be due to the difference in study design, population (age and sex), and the antibody detection technique. In a study by Barin et al., the CoronaVac, another inactivated vaccine, induced the lowest seropositivity and anti-RBD IgG, followed by the ChAdOx1 nCoV-1 vaccine, with the BNT162b2 vaccine inducing the highest response. CoronaVac was also associated with the highest rate of antibody decline, followed by the ChAdOx1 nCoV-1 vaccine [12].

Unlike the RBD, S2 is more conserved across coronaviruses, and mutations are less likely in this subunit [13,14]. Antibodies against these conserved regions can effectively neutralize the virus, conferring protective immunity to support the recovery of patients and constituting an effective method for passive therapy [15,16]. In our study, the level of the anti-S2 antibody was significantly higher in the ChAdOx1 nCoV-1 group than in the BBIBP-CorV group; however, this was not associated with higher protection against infection after ChAdOx1 nCoV-1 vaccination. Long-term follow-up is needed to evaluate the role of this antibody in protecting against COVID-19. In the study from Jordan, although the number of reinfections was higher in the BBIBP-CorV group, the post-vaccination infection occurred earlier, on average, in the ChAdOx1 nCoV-1 group (59.3 days) than in the BBIBP-CorV group (78.5 days) [10]. Our data also show that the median time until infection was shorter in the ChAdOx1 nCoV-1 group.

Our study revealed a significant increase in the levels of antibodies after the second dose of the ChAdOx1 nCoV-1 vaccine compared with the first dose, where the mean titers of antibodies against all SARS-CoV-2 proteins were significantly elevated after the second dose of the ChAdOx1 nCoV-19 vaccine compared with the first dose, except for those against S2 and nucleocapsid proteins. Folegatti et al. similarly showed that homologous boosting with two doses of the same vaccine was associated with increased antibody responses [9]. Other studies have also reported that anti-spike and anti-RBD antibodies were lower after the first dose of the ChAdOx1 nCoV-1 vaccine than after mRNA vaccines but increased significantly after heterologous vaccination with an mRNA vaccine booster [17,18]. In a study by Kittikraisak et al., the anti-S1 antibody level dropped below the positive cutoff within two months among those who received one dose of the ChAdOx1 nCoV-1 vaccine compared with about four months among those who received two doses of the BBIBP-CorV vaccine [19]. Collectively, our results support the need for booster doses to protect against SARS-CoV-2 infection.

Generally, adenovirus-vectored vaccines are known to induce strong cellular immunity. To evaluate the cell-mediated immune response after vaccination, we compared the percentages of T cells and B cells as well as the T cell subsets (CD4 and CD8). Although the T cell percentage was significantly lower in the ChAdOx1 nCoV-1 group, the percentage of CD8^+^ cells was significantly higher, indicating a shift toward a cytotoxic T cell immune response in this group. Different studies showed that the ChAdOx1 nCoV-1 vaccine induces a robust T cell response against both S1 and S2 antigens, Spike-specific effector T cell responses appeared one week after vaccination, and the response was predominantly Th1 [9,20].

Interestingly, an increased percentage of CD4^+^ cells in our study was associated with increased levels of different antibodies, especially anti-S1, anti-S2, and anti-RBD, implicating these cells in the development of different antibodies after both types of vaccinations. Deng et al. demonstrated that the BBIBP-CorV vaccine was associated with specific T cell responses to different SARS-CoV-2 proteins (S, N, and E) [21]. A study by Liu et al. showed that, although the level of neutralizing antibodies elicited by two doses of the BBIBP-CorV vaccine dropped five months after the second dose, spike-specific memory T and B cells were detectable. After the third dose, these cells formed the basis for a quick humoral and cell-mediated immune response and offered durable protection [22].

Although some participants in our study were infected after vaccination, none were admitted to the hospital or contracted a severe form of COVID-19, indicating that both vaccines effectively protected against mortality in the short term. The attenuation of the risk of COVID-19-related hospitalization was similar to other published studies on the ChAdOx1 nCoV-1 vaccine [23,24].

The side effects after BBIBP-CorV vaccination were mild, similar to the clinical trials of this vaccine, which reported no adverse events during the 4 weeks after vaccination [25,26]. On the other hand, several cases of thrombocytopenia and thrombotic events have been reported after vaccination with ChAdOx1 nCov-19 or ChAdOx1 nCoV-1 [27]. However, none of these side effects were reported in our cohort, and moderate side effects after vaccination were tolerable, similar to early clinical trials of this vaccine, wherein most adverse events were mild or moderate and self-limiting [9].

## 5. Conclusions and Recommendations

Our study shows that the BBIBP-CorV vaccine elicits an immune response and short-term protection against infection that is comparable to the ChAdOx1 nCoV-1 vaccine. These results have important implications for policymakers, especially in developing countries. Inactivated vaccines are easier to manufacture in these countries if the facilities are available. Egypt has manufactured one billion doses a year of China’s Sinovac vaccine, becoming the biggest vaccine producer in the Middle East and Africa [28]. Moreover, the BBIBP-CorV vaccine is accompanied by only a few side effects, which may limit absences from work and lead to better economic benefits. Follow-up is needed to study the long-term protective effects of both types of vaccines included in this work.

## 6. Limitations of the Study

Our study has several limitations. First, we compared only two types of vaccines, which were commonly administrated in the early vaccination phase in Egypt. Our study did not include other types of vaccines, especially mRNA-based ones. Second, we did not identify or study the efficacy of these vaccines against different variants that emerged during the pandemic. Third, our panel to identify T and B cell subsets was limited and did not identify specific immune cells against the virus. Finally, we did not evaluate the cell-mediated and humoral immunity during the follow-up period. Accordingly, we did not compare the immune response with protection against infection risk.

## Figures and Tables

**Table 1:** Demographic and clinical characteristics of the participants (n = 109).

Factors	ChAdOx1 nCoV-19 (n = 60)		BBIBP-CorV (n = 49)		*p*
Sex (F), n (%)	38	63.33	32	65.31	0.83 ^†^
Age, mean (SD)	37.46	8.72	45.42	9.09	**<0.001** ^§^
Comorbidity (Yes), n (%)	2	3.33	15	30.61	**<0.001** ^†^
Smoking (Yes), n (%)	2	3.33	2	4.08	0.81 ^†^
History of COVID-19 (Yes), n (%)	28	46.67	14	28.57	**0.05** ^†^
History of Contact (Yes), n (%)	26	43.33	31	63.27	**0.03** ^†^

^§^: Independent *t*-test, ^†^: Chi-squared test, *p* < 0.05 considered significant. Bold values are the statistically significant values.

**Table 2:** Humoral immune response after the first dose of the ChAdOx1 nCoV-1 vaccine and the second dose of both vaccines (n = 109).

Vaccine Type	Anti-Spike	Anti-S1	Anti-S2	Anti-RBD	Anti-Nucleocapsid
	n (%)	n (%)	n (%)	n (%)	n (%)
**ChAdOx1 nCoV-19-1st (n = 60)**	35 (58.3)	28 (46.6)	26 (43.3)	31 (51.6)	4 (6.6)
**ChAdOx1 nCoV-19-2nd (n = 60)**	51 (85)	49 (81.6)	51 (85)	51 (85)	12 (20)
**BBIBP-CorV-2nd (n = 49)**	46 (93.8)	44 (89.8)	32 (65.3)	46 (93.8)	17 (34.6)
*p* *	**<0.001**	**<0.001**	**<0.001**	**<0.001**	**0.001**

* Test of significance: chi-squared test, *p* < 0.05 was considered significant. Bold values are the statistically significant values.

**Table 3:** Cell-mediated immune response after vaccination (n = 109).

Factors	ChAdOx1 nCoV-19 (n = 60)		BBIBP-CorV (n = 49)		*p* ^§^
	Mean	SD	Mean	SD	
T cells (CD3+)	54.8	15.8	59.5	12	0.15
B cells (CD19+)	8.55	3.21	8.11	2.96	0.51
CD4+ cells	70.1	19.3	73.82	9.57	0.28
CD8+ cells	31.7	14.6	25.08	9.31	**0.02**

^§^: Independent *t*-test, *p* < 0.05 was considered significant. All expressions were estimated by the level of (percentage) from lymphocytes, CD4, and CD8 cell subsets from T lymphocytes. Bold values are the statistically significant values.

**Table 4:** Factors affecting cell-mediated immune response after vaccination.

Factors	T Cells (%)		B Cells (%)		CD4+ (%)		CD8+ (%)	
	Coef	*p*-Value	Coef	*p*-Value	Coef	*p*-Value	Coef	*p*-Value
Total number of positive antibodies	−6.35	**0.018**	−2.116	0.016	13.32	**0.002**		
Vaccine (BBIBP-CorV)	**Reference**							
Vaccine (ChAdOx1 nCoV-1)	−5.09	**0.026**		-	-		6.77	0
Contact to COVID-19 cases (Yes)	**Reference**							
Contact to COVID-19 cases (No)	5.13	**0.012**	-	-	5.84	**0.011**	−3.66	**0.035**
Anti-Spike (+ve)	**Reference**							
Anti-Spike (−ve)	−10.99	**0.005**	-	-	-	-	-	-
Anti-S1 (+ve)	**Reference**							
Anti-S1 (−ve)	-	-	−2.66	0.024	12.07	**0.034**	-	-
Anti-S2 (+ve)	**Reference**							
Anti-S2 (−ve)	−7.09	0.074	−2.063	0.014	12.65	**0.003**	-	-
Anti-RBD (+ve)	Reference							
Anti-RBD (−ve)	8.17	**0.019**	-	-	5.98	0.058	−6.18	**0.001**
Anti-nucleocapsid (+ve)	Reference							
Anti-nucleocapsid (−ve)	−5.78	**0.031**	−1.547	0.012	-	-	-	-

General linear model with stepwise elimination methods, Coef.: coefficient, the sign before coefficient denotes the direction of the relationship, *p* < 0.05 was considered significant. Bold values are the statistically significant values.

**Table 5:** Side effects of vaccination (n = 109).

Factor	ChAdOx1 nCoV-1 (n = 60)		BBIBP-CorV (n = 49)		*p*
	Mean	Range	Mean	Range	
Total number of side effects	1.35	(0–4)	0.78	(0–3)	**0.01** ^§^
Degree of side effects					
	N	%	N	%	
No	22	36.67	24	48.98	**<0.001** ^†^
Mild	10	16.67	19	38.78	
Moderate	28	46.67	6	12.24	
Fever (Yes)	23	38.33	1	2.04	**<0.001** ^†^
Fatigue (Yes)	26	43.33	13	26.53	0.06 ^†^
Body pain (Yes)	17	28.33	5	10.2	**0.01** ^†^
Headache (Yes)	10	16.67	7	14.29	0.73 ^†^
Pain at injection site (Yes)	5	8.33	12	24.49	**0.02** ^†^

Continuous data presented as mean and range, and categorical data as number and percentage. ^§^: Independent *t*-test, ^†^: Chi-squared test, *p* < 0.05 was considered significant. Bold values are the statistically significant values

**Table 6:** Short-term protective effects of vaccination.

Factors	ChAdOx1 nCoV-1 (n = 60)		BBIBP-CorV (n = 49)		*p*
	N	%	N	%	
**Post-vaccine infection**	25	41.6	22	44.9	0.26 ^†^
**Degree of severity**	N	%	N	%	
**No**	41	68.4	27	55.1	0.3 ^†^
**Mild**	13	21.6	14	28.57	
**Moderate**	6	10	8	16.33	
	Median	IQR	Median	IQR	
**Duration after vaccination (Month)**	1	(1–2)	2	(1–3.5)	**0.01** ^§§^

^§§^: Kruskal–Wallis test, ^†^: Chi-squared test, *p* < 0.05 was considered significant. Bold values are the statistically significant values.

## Data Availability

The data presented in this study are available upon request from the corresponding author.
